# The Naked Mole Rat Genome Resource: facilitating analyses of cancer and longevity-related adaptations

**DOI:** 10.1093/bioinformatics/btu579

**Published:** 2014-08-28

**Authors:** Michael Keane, Thomas Craig, Jessica Alföldi, Aaron M. Berlin, Jeremy Johnson, Andrei Seluanov, Vera Gorbunova, Federica Di Palma, Kerstin Lindblad-Toh, George M. Church, João Pedro de Magalhães

**Affiliations:** ^1^Integrative Genomics of Ageing Group, Institute of Integrative Biology, University of Liverpool, Liverpool, UK, ^2^Broad Institute of MIT and Harvard, Cambridge, MA, USA, ^3^Department of Biology, University of Rochester, NY, USA, ^4^Vertebrate and Health Genomics, The Genome Analysis Center, Norwich, UK, ^5^Department of Medical Biochemistry and Microbiology, Science for Life Laboratory, Uppsala University, Uppsala, Sweden and ^6^Department of Genetics, Harvard Medical School, Boston, MA, USA

## Abstract

**Motivation:** The naked mole rat (*Heterocephalus glaber*) is an exceptionally long-lived and cancer-resistant rodent native to East Africa. Although its genome was previously sequenced, here we report a new assembly sequenced by us with substantially higher N50 values for scaffolds and contigs.

**Results:** We analyzed the annotation of this new improved assembly and identified candidate genomic adaptations which may have contributed to the evolution of the naked mole rat’s extraordinary traits, including in regions of p53, and the hyaluronan receptors CD44 and HMMR (RHAMM). Furthermore, we developed a freely available web portal, the Naked Mole Rat Genome Resource (http://www.naked-mole-rat.org), featuring the data and results of our analysis, to assist researchers interested in the genome and genes of the naked mole rat, and also to facilitate further studies on this fascinating species.

**Availability and implementation:** The Naked Mole Rat Genome Resource is freely available online at http://www.naked-mole-rat.org. This resource is open source and the source code is available at https://github.com/maglab/naked-mole-rat-portal.

**Contact:** jp@senescence.info

## 1 INTRODUCTION

The naked mole rat (NMR; *Heterocephalus glaber*) is a long-lived subterranean rodent native to the Horn of Africa. It can not only live to >30 years, making it the longest-lived rodent, but is also extremely resistant to neoplasia (Buffenstein, 2008; Tian *et al.*, 2013), and as a result is an ideal model for research on longevity, cancer and disease resistance. The NMR genome was sequenced at the BGI in 2011 to 92-fold coverage with a contig N50 of 19.3 kb and scaffold N50 of 1.6 Mb (Kim *et al.*, 2011). Here we describe a higher quality assembly (HetGla_female_1.0), which has subsequently been sequenced by us at the Broad Institute, its analysis and availability on a purpose-built portal, the Naked Mole Rat Genome Resource (http://www.naked-mole-rat.org).

## 2 METHODS

Briefly, high molecular weight DNA was extracted from tissues of a single partially inbred female adult NMR obtained from the colony established by Vera Gorbunova at the University of Rochester, USA. The founder animals originated from the colony of J.U. Jarvis, at the University of Cape Town, South Africa. The *Heterocephalus glaber* assembly, HetGla_female_1.0, was constructed from 180 bp paired end fragment libraries (45 × coverage), 3 kb jumping libraries (42 × coverage), 6–14 kb sheared jumping libraries (2 × coverage) and 40 kb FOSILLs (Williams *et al.*, 2012) (1 × coverage). All libraries were sequenced by Hi-Seq Illumina machines, producing 101 bp paired-end reads. Assembly of the NMR genome was carried out using the software program ALLPATHS-LG (Gnerre *et al.*, 2011) version R38830 with default parameters.

## 3 RESULTS

HetGla_female_1.0 has substantially higher N50 for contigs (47.8 kb) and scaffolds (20.5 Mb) when compared with the Kim *et al.* assembly (Table 1). NG50 values, based on a C-value of 2.9 pg (source: http://www.genomesize.com/result_species.php?id=4474), are also considerably higher for HetGla_female_1.0: 35.3 kb for contigs (versus 18.1 kb for the Kim *et al.* assembly) and 20.0 Mb for scaffolds (versus 1.5 Mb).

**Table 1.** Global statistics of the HetGla_female_1.0 (alias: hetGla2) assembly in comparison with the Kim *et al.* assembly (HetGla_1.0)

Statistics	hetGla2	HetGla_1.0
RefSeq Assembly ID	GCF_000247695.1	GCF_000230445.1
Total sequence length	2 618 204 639	2 643 961 837
Number of scaffolds	4229	39 266
Scaffold N50	20 532 749	1 603 177
Number of contigs	114 653	273 990
Contig N50	47 778	21 750

To assist researchers in studying the genome and genes of the NMR to improve understanding of its extraordinary traits, and also to foster further studies employing this fascinating species, we developed a freely available web portal, the Naked Mole Rat Genome Resource (http://www.naked-mole-rat.org). Our portal features an annotation of the HetGla_female_1.0 assembly generated by the NCBI using the NCBI Eukaryotic Genome Annotation Pipeline (http://www.ncbi.nlm.nih.gov/books/NBK169439/). To assess the accuracy of this annotation, 4578 proteins were identified which exhibit at least 99% length conservation between human, mouse, rat and guinea pig orthologs. Of these, 3413 exceed the same 99% length threshold using the annotation of the HetGla_female_1.0 assembly, compared with 2158 using the annotation of Kim *et al.*

All annotated NMR sequences derived from the NCBI annotation of HetGla_female_1.0 are available on our portal: 42 117 coding sequences, 1779 non-coding sequences and 41 963 proteins. The 12 837 best-match NMR transcripts were identified based on coding sequence length similarity with the guinea pig ortholog, for which protein alignments and Ka/Ks ratios, calculated with the CodeML program of the PAML package v3.14 (Yang, 2007) using default parameters and guinea pig, mouse, rat and human orthologs, are also included on the portal. Genes that have been associated with longevity are cross-linked with the GenAge database (http://www.naked-mole-rat.org/annotations/results/genage/) (Tacutu *et al.*, 2013). A BLAST interface is also provided to allow users to quickly and easily search for sequences of interest (including coding and non-coding sequences, proteins and scaffolds). We have previously also sequenced the NMR transcriptome, which allowed us to compare liver gene expression profiles between NMRs and wild-derived mice (Yu *et al.*, 2011). The data and results of this work can also be downloaded (http://www.naked-mole-rat.org/static/downloads/RNA_seq_supplements.zip). Moreover, an additional ∼23-fold coverage assembly of the NMR genome generated by The Genome Analysis Centre (TGAC) based on two Illumina paired-end sequencing runs is available for download (http://www.naked-mole-rat.org/static/downloads/naked_mole_rat_contigs.zip).

Guinea pig genes were used to analyse NMR orthologs of potential significance because it is the most closely related species with a high coverage genome. Functionally enriched DAVID (v6.7) clusters (using human/mouse orthologs and a background of the 12 837 best-match transcripts; otherwise default parameters were used) with an enrichment score >1.3, corresponding to *P* < 0.05 (Huang *et al.*, 2009), for the top 5% of NMR genes by Ka/Ks included cytokine activity, signal peptide and defense response (Table 2).

**Table 2.** DAVID clusters of the highest-ranked genes by Ka/Ks between NMR and guinea pig

Cluster	Enrich. Score	No. genes	No. annots.
Signal peptide	11.86	165	10
Cytokine	10.53	36	4
Defense response	9.87	50	3
Immunoglobin domain	6.35	34	7
Cell surface	6.19	28	3

**Fig. 1.**
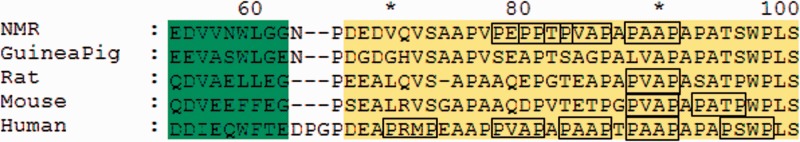
Alignment of p53 sequences from NMR, guinea pig, rat, mouse and human. The TADII is in green, the PRD in yellow and PXXP motifs are boxed

Given the higher quality of this more recent genome annotation, we assessed whether we could identify novel candidate genes in the NMR that were not detected by Kim *et al.* In particular, because p53 substitutions identical to those found in human tumours have been identified in the related blind mole rat *Spalax ehrenbergi* (Ashur-Fabian *et al.*, 2004), it is relevant to assess whether there is any evidence of adaptive evolution in NMR p53. While the NMR p53 coding sequence is, not surprisingly, subject to purifying selection (Ka/Ks = 0.26), a window from codons 41–80 was observed, encompassing transactivation domain 2 (TADII) and most of the proline-rich domain (PRD), which had a signature of positive selection (Ka/Ks = 2.19). The PRD is found between residues 58–98 and 55–95 of the human and mouse proteins, respectively (Walker and Levine, 1996). The human PRD contains numerous prolines including five PXXP (P = proline, X = any amino acid) motifs, compared with only two in mouse and one in rat (Toledo *et al.*, 2007). Interestingly, the NMR PRD substitutions include four proline residues, resulting in an additional four PXXP motifs relative to the guinea pig domain (Fig. 1).

This raises the possibility of convergent evolution of additional prolines and PXXP motifs in the p53 PRDs of humans and NMRs, two species which evolved an extended lifespan and consequent requirement for an enhanced DNA damage response. In addition, there are two NMR substitutions in the 9aaTADII, which has been reported to mediate apoptosis by activating targets, including *MDM2* and *BAX* (Zhu *et al.*, 1998).

Numerous proteins have been shown to interact with p53, including *BRCA1* via a region from residues 224–500 (Zhang *et al.*, 1998). There is a strong signal of selection within this region of NMR *BRCA1*, particularly from codons 430–470 (Ka/Ks > 10), which may influence the interaction with p53.

Early contact inhibition (ECI) has been identified as a novel anti-cancer mechanism in the NMR (Seluanov *et al.*, 2009), with high-molecular mass hyaluronan as the extracellular signal, which is partly transmitted via the *CD44* receptor (Tian *et al.*, 2013). Interestingly, a signal of selection (Ka/Ks > 1) was observed not only in *CD44*, from guinea pig codons 401–440, 501–540 and 661–700, but also in another hyaluronan receptor, *HMMR (RHAMM)*, from codons 321–360, 381–420 and 441–480, suggesting that it may also contribute to transmission of the ECI signal.

**Fig. 2.**
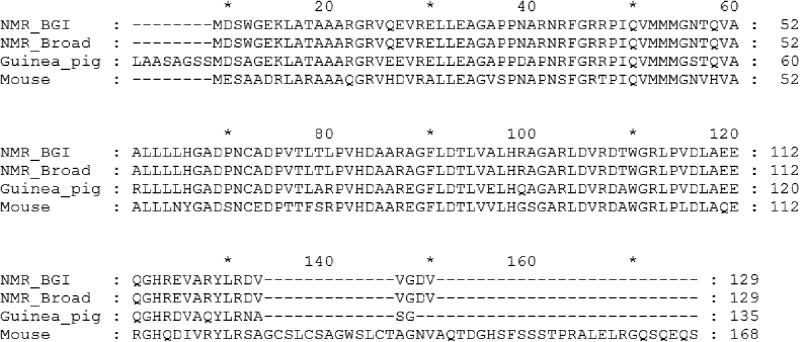
Alignment of p16 sequences from guinea pig, mouse and NMR using both the Broad and BGI assemblies

Kim *et al.* reported that relative to mice, two early stop codons in the NMR p16^Ink4a^ transcript were predicted to produce a truncated protein. There are no *Cdkn2a* transcripts in the NCBI annotation; however, a predicted transcript was generated based on alignments of the mouse and guinea pig exons with the assembly and transcriptome. Although there are no significant differences with the transcript predicted by Kim *et al.*, it is important to note that the guinea pig protein is also of similar length and shorter than in mice, indicating that this is not an NMR-specific adaptation (Fig. 2).

In conclusion, we have developed a NMR portal using a genome assembly of superior quality for the research community to benefit from this data. Our portal is designed so it can be easily updated if the NMR genome annotation is updated in the future. We also performed a reanalysis of the NMR genome using this improved assembly, which revealed further candidate genes of potential relevance to adaptive changes in the context of aging and cancer. We hope this research will facilitate and encourage studies in these amazing animals.
